# Prevalence of toxoplasmosis and genetic characterization of *Toxoplasma gondii* strains isolated in wild birds of prey and their relation with previously isolated strains from Turkey

**DOI:** 10.1371/journal.pone.0196159

**Published:** 2018-04-18

**Authors:** Muhammet Karakavuk, Duygu Aldemir, Aurélien Mercier, Esra Atalay Şahar, Hüseyin Can, Jean-Benjamin Murat, Ömer Döndüren, Şengül Can, Hüseyin Gökhan Özdemir, Aysu Değirmenci Döşkaya, Bayram Pektaş, Marie-Laure Dardé, Adnan Yüksel Gürüz, Mert Döşkaya

**Affiliations:** 1 Department of Parasitology, Ege University Faculty of Medicine, Bornova, İzmir, Turkey; 2 Department of Internal Medicine, Faculty of Veterinary Medicine, Uludağ University Institute of Health Sciences, Görükle Campus, Nilüfer-Bursa, Turkey; 3 İzmir Wildlife Park, Municipality of İzmir, Sasalı, Çiğli, İzmir, Turkey; 4 Centre National de Référence (CNR) Toxoplasmose/ Toxoplasma Biological Resource Center (BRC), Centre Hospitalier-Universitaire Dupuytren and INSERM UMR 1094, Neuroépidémiologie Tropicale, Laboratoire de Parasitologie-Mycologie, Faculté de Médecine, Université de Limoges, Limoges, France; 5 Department of Biology, Molecular Biology Section, Ege University Faculty of Science, Bornova, İzmir, Turkey; 6 The Protection and Development Union of İzmir Bird Paradise, Konak, İzmir, Turkey; 7 Computer Research and Application Center, Manisa Celal Bayar University, Muradiye, Manisa, Turkey; 8 İzmir Atatürk Training and Research Hospital, Department of Microbiology, Yeşilyurt, İzmir, Turkey; NIH, UNITED STATES

## Abstract

*Toxoplasma gondii* is a protozoon parasite that causes congenital toxoplasmosis, as well as other serious clinical presentations, in immune compromised humans. Analyses of the prevalence and genotyping of strains from the definitive host and intermediate hosts will help to understanding the circulation of the different strains and elucidating the role of the genotype(s) in human toxoplasmosis. Turkey has a specific geographic location bridging Africa, Europe, and Asia. We hypothesized that *T*. *gondii* strains may have been transferred to Turkey from these continents via migratory birds or vice versa. The present study aimed to assess the prevalence of toxoplasmosis in wild birds of prey of İzmir and Manisa provinces as well as genetically characterize *T*. *gondii* strains from these wild birds to show the relation between bird strains and neighboring stray cats as well as human strains previously isolated in Turkey. Tissues obtained from 48 wild birds were investigated for the presence of *T*. *gondii* DNA and then bioassayed in mouse. Isolated strains were genotyped using 15 microsatellite markers. The prevalence of *T*. *gondii* DNA was found to be 89.6% (n: 43/48) in wild birds. Out of 43 positive samples, a total of 14 strains were genotyped by 15 microsatellite markers. Among them, eight were type II, three were type III and three were mixture of genotypes (two type II/II and one was II/III). These are the first data that showed the presence of *T*. *gondii* and types II and III genotypes in wild birds of Turkey. Moreover, Africa 1 was not detected. In addition, cluster analysis showed that *T*. *gondii* strains within type II and III lineage have close relation with strains previously isolated from stray cats in İzmir. Further studies are required to isolate more strains from human cases, other intermediate hosts, and water sources to reveal this relation.

## Introduction

*Toxoplasma gondii* is a protozoan parasite that causes congenital toxoplasmosis, as well as other serious clinical presentations in immune compromised humans. The parasite has also been recently linked to behavioral diseases in humans. *T*. *gondii* genotypes are being linked to some of these clinical presentations [1–5]. Strains are being classified into three major clonal lineages, types I, II, and III, and other additional lineages, as well as atypical and recombinant genotypes based on genetic polymorphism through the use of various molecular techniques [1–4, 6, 7]. Isolation and genotyping of strains from the definitive host felines, humans and other intermediate hosts will help better understand the circulation of the different strains both at a global and a local geographical scale. This may also help elucidate the role of the genotype(s) in human toxoplasmosis. Thus, genotyping *T*. *gondii* strains has utmost importance nowadays.

Certain genotypes predominate in specific geographic locations such as type II in Europe, [5], non-archetypal genotypes named Africa 1, 2, and 3 in addition to type II or III lineages in sub-Saharan Africa [3, 8, 9], type II and III strains in North Africa, the Middle East and the Arabic peninsula [10–12], the three major clonal lineages in addition to the predominant genotype, Chinese 1 in Asia [13–17], the three major lineages and recombinant strains in North and Central America, strains with high diversity in South America [18–20].

Our genotyping research started several years ago with the detection of Africa 1 genotype in two local congenital toxoplasmosis cases whose mothers lived in Turkey [21]. This was interesting since atypical Africa 1 genotype was only detected in animals and immunocompromised patients from sub-Saharan Africa [9, 21]. In our second research, we asked the question what were the prevalent genotypes in definitive host felines in Turkey. Twenty-two isolates were isolated from 100 deceased stray cats of İzmir, Turkey: 19 were type II (86.3%), two were type III (9%), and one was Africa 1 genotype (4.5%) [22]. Therefore, in addition to Africa 1, two major clonal lineages were also present in stray cats of İzmir.

**Fig 1:**
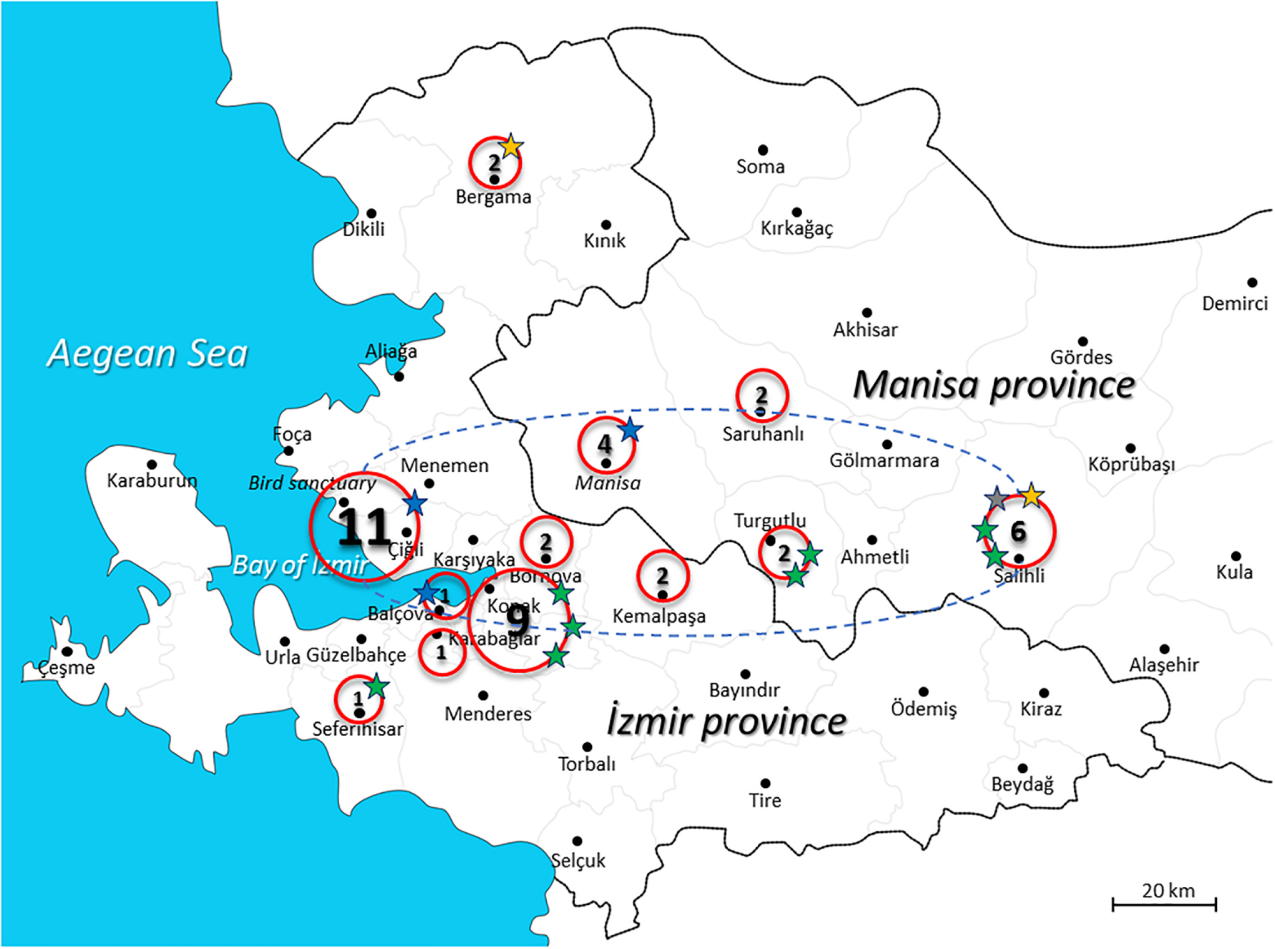
The geographic distribution of 43 wild birds in İzmir and Manisa provinces and the distribution of genotypes detected. Locations of five wild birds were unknown. A possible route of migration appears in Gediz lowland between İzmir and Manisa provinces is represented by dotted lane. Gediz lowland is connected to İzmir Bird Sanctuary, a popular stop point for resident and migratory birds of prey. Green and blue stars represent type II and III lineages, respectively. Mix of two type II strains is represented by orange star. Mix of type II and III strains is represented by grey star.

Turkey has a specific geographic location bridging Africa, Europe, and Asia and *T*. *gondii* strains may have been transferred between these continents via stray cats or other animals, including migratory birds [22]. İzmir is the third biggest city in Turkey located close to the Western Anatolia and has a huge wild life park with bird sanctuary (Fig 1). Stray cats can easily enter the wild life park or bird sanctuary and get into close relation with local cats living in this area which can easily capture migratory birds or eat carcasses of them. Thus, this specific location aroused the following question: can the migratory wild birds of İzmir harbor the genotypes detected in humans and stray cats? and can there be more different strains in wild life of İzmir, Turkey? Therefore, the present study aimed to assess the prevalence of *Toxoplasma* infection, isolate and genetically characterize *T*. *gondii* strains in wild birds of prey of İzmir and Manisa provinces. In addition, clustering analyses was performed to show the relation of bird and neighboring cat strains isolated as well as human strains previously isolated in Turkey.

## Materials and methods

### Ethics statement

Experiments performed with animals were performed under the instructions and approval of the Institutional Animal Care and Use Committee (IACUC) of Ege University for animal ethical norms (Permit number: 2014–016).

During the experiments 6–8 week old female *Swiss outbred* mice, obtained from the Bornova Veterinary Control Institute Animal Production Facility were housed under standard and suitable conditions. Animals were checked for humane endpoints every day such as rapid weight loss (>~20% of gross body weight), inability to drink water or eat food, or loss of elasticity in skin indicative of dehydration. In any of these circumstances, we pre-euthanized the animals with ketamine hydrochloride (2 mg/kg) and 2% xylazine (3 mg/kg) and then euthanized with cervical dislocation.

Deceased birds were found in İzmir Bird Sanctuary (i.e. İzmir Bird Paradise) or brought to İzmir Wildlife Park Clinics from various districts of Izmir and Manisa provinces located in western Anatolia.

### Wild birds and sample collection

Wild birds were found in the İzmir Bird Sanctuary or brought to İzmir Wildlife Park Clinics from eight different districts of İzmir province (Çiğli, Konak, Bornova, Bergama, Kemalpaşa, Balçova, Karabağlar, and Seferihisar) and four different districts from Manisa province (Saruhanlı, Manisa, Turgutlu, Salihli) (Fig 1). The permission to collect samples were issued by Ministry of Forestry and Water Management, General Directorate of Nature Conservation and National Parks as well as Municipality of Izmir, İzmir Wildlife Park Branch Directorate. The sampling procedures were reviewed as part of obtaining the field permit.

İzmir Wildlife Park and İzmir Bird Sanctuary are located in Çiğli district. Tissue (brain and heart) samples were collected from deceased birds (n: 48). The species of wild birds were composed of common buzzard (*Buteo buteo*) (n: 25), Eurasian sparrowhawk (*Accipiter nisus*) (n: 5), barn owl (*Tyto alba*) (n: 2), yellow-legged gull (*Larus michahellismichahellis*) (n: 2), Eurasian eagle-owl (*Bubo bubo*) (n: 2), thrush nightingale (*Luscinia luscinia*), wood pigeon (*Columba palumbus*), little owl (*Athene noctua*), Eurasian stone curlew (*Burhinus oedicnemus*), white stork (*Ciconia ciconia*), great cormorant (*Phalacrocorax carbo*), dalmatian pelican (*Pelecanus crispus*), greater flamingo (*Phoenicopterus roseus*), peregrine falcon (*Falco peregrinus*), black stork (*Ciconia nigra*), common kestrel (*Falco tinnunculus*), and little tern (*Sternula albifrons*) (Table 1).

**Table 1:** Microscopy, Real Time PCR, and genotyping results of *T*. *gondii* strains isolated from wild birds of İzmir and Manisa provinces.

No ^a^	Name of the isolate ^b^	Name of the bird	Predator bird	Movement patterns [23] Local resident (L)/Partially migratory (PM) ^C^/Migratory (M)	Location of the isolate	Cause of death	Real Time PCR CP_*T*_ value (bird tissue) ^d^^,^^e^	Microscopy ^f^ (mouse brain)	Live/Toxoplasmic DNA extracts	Genotype
1	TgBirdTr_Izmir 1	Barn owl (*Tyto alba*)	Pr	L	Çiğli (IWP)	Unknown Trauma (Fracture of wing)	27.47	N	DNA extract	Type III
2	TgBirdTr_Izmir 2	Yellow-legged gull (*Larus michahellis*)	Pr	PM (wintering in North Africa, European coast, south-west Asia)	Konak	Unknown Trauma (Fracture of wing)	30.01	N	DNA extract	Type II
3	-	Eurasian eagle-owl (*Bubo bubo*)	Pr	L	Kemalpaşa	Paralysis	31.62	N	DNA extract	-
4	-	Thrush nightingale(*Luscinia luscinia*)	-	M (wintering in Africa)	Konak	Diarrhea	30.87	N	DNA extract	-
5	-	Wood pigeon (*Columba palumbus*)	-	M (wintering in Europe)	Konak	Diarrhea	32.58	N	DNA extract	-
6	TgBirdTr_Izmir 3	Yellow-legged gull (*Larus michahellis*)	Pr	PM (wintering in North Africa, European coast, south-west Asia)	Balçova	Trauma (Car crash)	33.76	P	Live	Type III
7	-	Little owl (*Athene noctua*)	Pr	L	Çiğli (IBS)	Paralysis	35.28	N	DNA extract	-
8	-	Common buzzard (*Buteo buteo*)	Pr	PM (wintering in Africa and Southern Asia)	Çiğli (IWP)	Paralysis	30.64	N	DNA extract	-
9	-	Eurasian Thick-knee, Stone Curlew (*Burhinus oedicnemus*)	Pr (insects)	M (wintering in southern Europe, the Middle East and Africa)	Çiğli (Kaklıç)	Paralysis	34.79	N	DNA extract	-
10	-	White stork (*Ciconia ciconia*)	Pr (Fish, insects, reptiles etc.)	M (wintering in Africa)	Konak	Unknown Trauma (Fracture of wing and beak)	32.14	N	DNA extract	-
11	TgBirdTr_Izmir 4	Common buzzard (*Buteo buteo*)	Pr	PM (wintering in Africa and southern Asia)	Seferihisar	Unknown Trauma (Fracture of wing)	28.2	P	Live	Type II
12	-	Common buzzard (*Buteo buteo*)	Pr	PM (wintering in Africa and southern Asia)	Manisa (Muradiye)	Unknown Trauma (Fracture of wing)	29.96	N	DNA extract	-
13		Great cormorant (*Phalacrocorax carbo*)	Pr (Fish)	PM (wintering in Africa, southern Asia, Australia)	Çiğli (IWP)	Poaching	29.05	P	Live	-
14	-	Common buzzard (*Buteo buteo*)	Pr	PM (wintering in Africa and southern Asia)	Manisa	Unknown Trauma (Fracture of wing)	26.16	N	DNA extract	-
15	TgBirdTr_Manisa 1	Common buzzard (*Buteo buteo*)	Pr	PM (wintering in Africa and southern Asia)	Manisa	Trauma (Fracture of leg)	25.32	N	DNA extract	Type III
16	TgBirdTr_Izmir 5	Barn owl (*Tyto alba*)	Pr	L	Konak	Unknown Trauma (Fracture of wing)	25.47	P	Live	Type II
17	-	Common buzzard (*Buteo buteo*)	Pr	PM (wintering in Africa and southern Asia)	Kemalpaşa	Unknown Trauma (Fracture of wing)	29.9	N	DNA extract	-
18	-	Common buzzard (*Buteo buteo*)	Pr	PM (wintering in Africa and southern Asia)	Çiğli	Keratitis	30.86	N	DNA extract	-
19	-	Eurasian sparrowhawk (*Accipiter nisus*)	Pr	PM (wintering in southern Europe, southern Asia, Africa)	Salihli	Unknown Trauma (Fracture of wing)	34.00	N	DNA extract	-
20	-	Common buzzard (*Buteo buteo*)	Pr	PM (wintering in Africa and southern Asia)	Manisa	Unknown Trauma (Fracture of wing)	N	-	-	-
21	-	Common buzzard (*Buteo buteo*)	Pr	PM (wintering in Africa and southern Asia)	Salihli	Unknown Trauma (Fracture of wing)	N	-	-	-
22	-	Eurasian sparrowhawk (*Accipiter nisus*)	Pr	PM (wintering in southern Europe, Southern Asia, Africa)	Çiğli	Unknown Trauma (Fracture of wing)	N	-	-	-
23	-	Dalmatian pelican (*Pelecanus crispus*)	Pr (Fish)	PM (wintering in Eastern Europe, Asia, Nile river)	Çiğli (IBS)	Unknown Trauma (Fracture of leg)	24.25	N	DNA extract	-
24	TgBirdTr_Manisa 2	Common buzzard (*Buteo buteo*)	Pr	PM (wintering in Africa and southern Asia)	Turgutlu	Trauma (Car crash)	23.89	N	DNA extract	Type II
25	TgBirdTr_Izmir 6	Common buzzard (*Buteo buteo*)	Pr	PM (wintering in Africa and southern Asia)	Bergama	Unknown Trauma (Fracture of wing)	25.31	N	DNA extract	Mix (Type II/II)
26	-	Common buzzard (*Buteo buteo*)	Pr	PM (wintering in Africa and southern Asia)	Çiğli (IWP)	Systemic infection	24.8	N	DNA extract	-
27	TgBirdTr_Manisa 3	Common buzzard (*Buteo buteo*)	Pr	PM (wintering in Africa and southern Asia)	Salihli	Keratitis	24.32	P	Live	Type II
28	-	Common buzzard (*Buteo buteo*)	Pr	PM (wintering in Africa and southern Asia)	Bornova	Unilateral Keratitis	24.81	N	DNA extract	-
29	-	Common buzzard (*Buteo buteo*)	Pr	PM (wintering in Africa and southern Asia)	-	Keratitis	25.81	N	DNA extract	-
30	-	Common buzzard (*Buteo buteo*)	Pr	PM (wintering in Africa and southern Asia)	-	Respiratory infection	25.87	N	DNA extract	-
31	TgBirdTr_Manisa 4	Eurasian eagle-owl (*Bubo bubo*)	Pr	L	Salihli	Respiratory infection	24.86	N	DNA extract	Mix (Type II/III)
32	TgBirdTr_Manisa 5	Common buzzard (*Buteo buteo*)	Pr	PM (wintering in Africa and southern Asia)	Salihli	Keratitis	25.24	N	DNA extract	Mix (Type II/II)
33	TgBirdTr_Manisa 6	Common buzzard (*Buteo buteo*)	Pr	PM (wintering in Africa and southern Asia)	Salihli	Unknown Trauma (Fracture of wing)	24.77	N	DNA extract	Type II
34	-	Common buzzard (*Buteo buteo*)	Pr	PM (wintering in Africa and southern Asia)	Bergama	Keratitis	27.26	N	DNA extract	-
35	-	Eurasian sparrowhawk (*Accipiter nisus*)	Pr	PM (wintering in southern Europe, Southern Asia, Africa)	Bornova	Keratoconjunctivitis	26.39	N	DNA extract	-
36	TgBirdTr_Manisa 7	Common buzzard (*Buteo buteo*)	Pr	PM (wintering in Africa and southern Asia)	Turgutlu	Hyphema	27.87	P	Live	Type II
37	-	Common buzzard (*Buteo buteo*)	Pr	PM (wintering in Africa and southern Asia)	Konak	Paralysis	27.60	N	DNA extract	-
38	-	Greater flamingo (*Phoenicopterus roseus*)	Pr (small invertebrates)	PM (Wintering in all Mediterranean basin and Africa)	Konak	Electric shock	28.95	N	DNA extract	-
39	-	Common buzzard (*Buteo buteo*)	Pr	PM (wintering in Africa and southern Asia)	Saruhanlı	Diarrhea	27.59	N	DNA extract	-
40	-	Eurasian sparrowhawk (*Accipiter nisus*)	Pr	PM (wintering in southern Europe, Southern Asia, Africa)	Karabağlar	Respiratory infection	26.04	N	DNA extract	-
41	TgBirdTr_Izmir 7	Common buzzard (*Buteo buteo*)	Pr	PM (wintering in Africa and southern Asia)	Konak	Paralysis	25.12	N	DNA extract	Type II
42	-	Eurasian sparrowhawk (*Accipiter nisus*)	Pr	PM (wintering in southern Europe, southern Asia, Africa)	Saruhanlı	Respiratory infection	28.07	N	DNA extract	-
43	-	Peregrine falcon (*Falco peregrinus*)	Pr	M (wintering in Africa)	Çiğli	Unknown Trauma (Fracture of wing)	31.83	N	DNA extract	-
44	-	Common kestrel (*Falco tinnunculus*)	Pr	PM (wintering in Africa)	Konak	Unknown Trauma (Fracture of wing)	N	-	-	-
45	-	Black stork (*Ciconia nigra*)	Pr (Fish, insects, reptiles etc.)	M (wintering in Africa)	-	Unknown Trauma (Fracture of wing)	31.19	N	DNA extract	-
46	-	Common buzzard (*Buteo buteo*)	Pr	PM (wintering in Africa and southern Asia)	-	Poaching	32.17	N	DNA extract	-
47	-	Common buzzard (*Buteo buteo*)	Pr	PM (wintering in Africa and southern Asia)	-	Diarrhea	31.26	N	DNA extract	-
48	-	Little tern (*Sternula albifrons*)	Pr (Fish)	M (wintering in Africa Arabian Peninsula, southeast Asia, and Australia)	Çiğli (IBS)	Incoordination, loss of voluntary control	N	-	-	-

^a^ Samples are numbered according to the date they have arrived to the laboratory.

^b^ The strains that could not be genotyped are not named.

^c^ migratory wild bird with local population.

^**d**^ Real time PCR was used to detect *T*. *gondii* DNA in mouse brains and cat tissues.

^**e**^ CP_*T*_ (Crossing point threshold). The amount of DNA is high when the value is low.

^f^ Microscopy detected the tissue cysts in mice brains.

N: negative; P: positive; Pr: Predator bird; L: Local bird; M: Migrating bird; IWP: İzmir Wildlife Park; IBS: İzmir Bird Sanctuary

### Bioassay in mice

Bird tissue homogenates were prepared from the brain and heart tissues as described [9, 22]. Initially, 10 gr tissue in 125 ml 0.9% NaCl was homogenized using a blender (Waring, USA). Then, tissue homogenate was added to a 500 ml Erlenmeyer flask and after adding 0.5 gr trypsin to the homogenate, the flask was incubated at 37°C with 120 rpm for 60 min using an incubator shaker (New Brunswick, USA). Next, the homogenate was filtered through sterile two layered gauze to 50 ml tubes and centrifuged at 910×g for 10 minutes. Thereafter, the supernatant was discarded and the pellet was washed two more times with 0.9% NaCl. After the last centrifugation, the pellet was resuspended with 5 ml 0.9% NaCl. A 500 μl aliquot was kept for DNA extraction and the remaining homogenate was incubated at 4°C overnight with Penicillin (40 U/ml)/Streptomycin (40 μg/ml) and Gentamycin (40 μg/ml). Next day, ~700 μl homogenate was inoculated intraperitoneally to mice (3 mice/group). The mice were closely monitored for 40 days and sacrificed afterwards. The brains of mice were removed aseptically and homogenized using a 5 ml syringe and 2 ml 0.9% NaCl. *T*. *gondii* tissue cysts and DNA were investigated in brain homogenates of mice using phase contrast microscopy (Nikon, USA) and Real Time PCR, respectively. Live strains were cryopreserved with RPMI medium containing a final concentration of 10% FCS and 10% DMSO [24].

### Real Time PCR

The presence of *T*. *gondii* DNA in wild birds or bioassayed mouse tissue samples were detected by Real Time PCR at the same day the tissue homogenate was prepared. Real Time PCR investigated *T*. *gondii* AF146527 gene as described [22, 25]. Isolation of DNA from the bird tissue or mouse brain homogenate was performed by QIAamp DNA mini kit (Qiagen, USA) according to the manufacturer’s protocol. During Real Time PCR, the primers were 5’-AGGCGAGGGTGAGGATGA-3’ (18nt, TOX-SE forward primer) and 5’-TCGTCTCGTCTGGATCGCAT-3’ (20nt, TOX-AS reverse primer) and the hybridization probes were 5’-GCCGGAAACATCTTCTCCCTCTCC-3’-FL (24nt, TOX FLU, labeled at the 3' end with fluorescein) and 5’-640-CTCTCGTCGCTTCCCAACCACG-3’ (22nt, TOX LCR labeled at the 5' end with LC-Red 640) (IDT).

Quantification and melting curve analysis was performed by 1.5 LightCycler Real Time instrument using LightCycler software, Version 3.5 (Roche). The PCR reaction with a 20 μl final volume included 1x LightCycler Fast Start DNA Master HybProbe mix with 5 mM MgCl_2_ (Roche), 5 μl purified DNA template or controls. The amplification reaction was performed as follows: 10 min initial denaturation step at 95°C, followed by 50 cycles of 5 seconds at 95°C, 10 seconds at 60°C, and 15 seconds at 72°C.

*T*. *gondii* genomic DNA serially 10-fold diluted ranging from 10^6^ to 10^1^ parasites/μl was used positive control and distilled water was used as negative control. In addition, melting curve analysis was performed as follows: 20 s denaturation step at 95°C (temperature transition rate 20°C/s) 20 s annealing step at 40°C (temperature transition rate 20°C/s) and an extension step that gradually increases the temperature to 85°C with a temperature transition rate of 0.2°C/s.

### Genotyping analysis

To genotype the strains, 15 microsatellite markers (*N61*, *B18*, *M33*, *M48*, *TUB2*, *N83*, *XI*.*1*, *N82*, *TgM-A*, *W35*, *IV*.*1*, *B17*, *N60*, *M102*, *AA*) located on 11 different chromosomes of *T*. *gondii* were amplified using a single multiplex PCR assay as described [26]. Initially, 25 μl final volume reaction included 12.5 μl multiplex PCR master mix (Qiagen), 5 μl DNA extracted from mouse brain or bird tissues, and 15 sets of primers (5 pmol each).

The amplification reaction was performed as follows: initial denaturation step at 95°C for 15 minutes, 35 cycles of 94°C for 30 seconds, 61°C for 3 minutes, and 72°C for 30 seconds, and a final extension at 60°C for 30 minutes. Next, PCR products were diluted 1/10 (mouse brain homogenate) or not diluted (bird tissue homogenate) using deionized formamide (Applied Biosystems). Then, 1 μl of diluted PCR product in deionized formamide was mixed with 0.5 μl DNA standard ROX 500 (Applied Biosystems) and 23.5 μl deionized formamide. Thereafter, the reaction mix was denatured at 95°C for 5 min and electrophoresed using an automatic sequencer (ABI PRISM 3130xl; Applied Biosystems). Microsatellite sizes were assessed using GeneMapper analysis software (Version 4.0; Applied Biosystems). As control, 15 reference strains belonging to type I (VAND, ENT, GT1,), type II (PRU, Me49), and type III (VEG, NED), atypical strains of Africa (DPHT, GAB3-GAL-DOM002, GAB5-GAL-DOM001, GAB3-GAL-DOM014, CCH002-NIA, and GAB2-GAL-DOM002), South America (GUY-CAN-FAM001 and TgCatBr5) and Turkey (Ankara and Ege-1 strains) were analyzed in parallel with strains isolated in this study [3, 9, 22, 26–28].

### Clustering analysis

A neighbor-joining tree containing bird strains isolated in İzmir and Manisa provinces of Turkey in addition to previously isolated strains from humans (Ankara and Ege-1), and stray cats in Turkey was constructed to quantify the extent of genetic distance between these strains and evaluate their position towards reference strains from different continents using Populations 1.2.30 (1999, Olivier Langella, CNRS UPR9034, http://bioinformatics.org/populations/). Trees were reconstructed using the Cavalli-Sforza and Edwards chord-distance estimator as described [29]. Thereafter, the analysis was repeated for 1000 bootstrap replicates in which loci were sampled with replacement. Unrooted trees were obtained with MEGA 6.05 software.

### Statistical analysis

Data obtained during the study were analyzed using Prism 3.03 (GraphPad, San Diego, CA) and a two-tailed unpaired t test with 95% confidence interval was used to determine the significance between the *T*. *gondii* DNA detected and undetected birds.

## Results

### Isolated *T*. *gondii* strains

Among the 48 wild bird samples, Real Time PCR of tissue homogenates was positive in 43 wild bird tissue homogenates and the prevalence of toxoplasmosis was 89.6%. All PCR positive wild bird tissues were bioassayed in mice. Six live strains were obtained by bioassay. Location details of PCR positive wild birds are shown in Table 1 and locations of five birds were unknown (Table 1).

### Microsatellite genotyping

Genotyping was performed on all PCR positive samples and lives strains. The success of genotyping was 32.5%, allowing to genotyping 14 strains over 43 positive DNA extracts. Among them, 6 live strains were isolated by both bioassay and PCR and remaining 8 strains were only toxoplasma DNA extracts (Table 1).

Among the 14 strains, 9 strains were genotyped by microsatellite analysis (15/15 MS markers), 2 were genotyped with 14/15 MS markers, 1 with 9/15 MS markers, 1 with 8/15 MS markers, and 1 with only 6/15 MS markers. This allowed identifying 8 type II isolates and three type III isolates (Table 2). In addition two samples contained a mix of two type II strains and one was a mixture of type II and type III strains, as evidence by the presence of 2 peaks for some markers. Remaining 29 positive samples were *Toxoplasma* DNA extracts from wild bird tissue samples and could not be genotyped (Table 1). The 14 strains were designated as TgBirdTr_Izmir and TgBirdTr_Manisa (Table 1). The isolates from İzmir were named TgBirdTr_Izmir 1–7. The remaining 7 isolates from Manisa were named TgBirdTr_Manisa 1–7.

**Table 2:** Genotyping results with 15 microsatellite markers of the 11 strains isolated from wild birds of İzmir and Manisa provinces with 17 reference *T*. *gondii* strains.

ISOLATE (GENOTYPE)	Microsatellite marker (size; base pair)^a^														
	*B18* (156–170)	*M33* (165–173)	*TUB2* (287–291)	*XI*.*1* (354–362)	*TgM-A* (203–211)	*W35* (242–248)	*IV*.*1* (272–282)	*B17* (334–366)	*M48* (209–243)	*M102* (164–196)	*N60* (132–157)	*N82* (105–145)	*AA* (251–332)	*N61* (79–123)	*N83* (306–338)
TgBirdTr_Manisa 1 (Type III)	160	165	289	356	205	242	278	336	213	190	147	111	269	89	312
TgBirdTr_Manisa 2 (Type II)	158	169	289	356	207	242	274	336	227	172	140	111	279	87	310
TgBirdTr_Manisa 3 (Type II)	158	169	289	356	207	242	274	336	211	176	138	109	275	87	312
TgBirdTr_Manisa 4 (mix of type III & II)	160	165	289	356	205	242	*274/278*	336	211	190	147	111	*261/267*	91	312
TgBirdTr_Manisa 5 (mix of two type II)	158	169	289	356	207	242	274	336	*211/213*	174	*140/142*	109	*261/291*	NA	310
TgBirdTr_Manisa 6 (Type II)	158	169	289	356	207	242	274	336	233	178	149	111	265	107	310
TgBirdTr_Manisa 7 (Type II)	158	NA	NA	356	207	242	NA	336	NA	178	NA	NA	NA	NA	NA
TgBirdTr_Izmir 1 (Type III)	160	165	289	356	205	242	278	336	213	190	147	111	267	89	312
TgBirdTr_Izmir 2 (Type II)	158	NA	289	NA	207	242	274	336	NA	NA	140	111	257	NA	NA
TgBirdTr_Izmir 3 (Type III)	160	165	289	356	205	242	278	336	213	190	147	111	275	89	312
TgBirdTr_Izmir 4 (Type II)	158	169	289	356	207	242	274	336	229	178	140	117	263	83	310
TgBirdTr_Izmir 5 (Type II)	158	169	289	356	209	242	274	336	213	174	140	127	263	103	314
TgBirdTr_Izmir 6 (mix of two type II)	158	169	289	356	207	242	NA	336	*213/221*	NA	NA	NA	NA	NA	NA
TgBirdTr_Izmir 7 (Type II)	158	169	289	356	207	242	274	336	NA	176	140	111	263	93	310
ANKARA (Africa 1)	160	165	291	354	205	248	274	342	227	166	147	111	295	91	310
EGE-1 (Africa 1)	160	165	291	354	205	248	274	342	227	166	149	111	289	91	310
DPHT (Africa 1)	160	165	291	354	205	248	274	342	225	166	147	111	271	89	306
GAB3-GAL-DOM014 (Africa 1)	160	165	291	354	205	248	274	342	229	166	142	111	271	95	306
GAB5-GAL-DOM001 (Africa 1)	160	165	291	354	205	248	274	342	231	166	149	111	277	87	306
GAB3-GAL-DOM002 (Africa 1)	160	165	291	354	205	248	274	342	223	166	147	111	269	89	306
CCH002-NIA (Africa 2)	160	165	289	354	205	248	274	336	225	166	145	111	273	89	308
GAB2-GAL-DOM002 (Africa 3)	160	165	291	354	207	242	278	342	223	166	142	111	277	97	310
ENT (Type I)	160	169	291	358	209	248	274	342	209	166	145	119	267	87	306
GT1 (Type I)	160	169	291	358	209	248	274	342	209	168	145	119	265	87	306
Me49 (Type II)	158	169	289	356	207	242	274	336	215	174	142	111	265	91	310
PRU (Type II)	158	169	289	356	207	242	274	336	209	176	142	117	265	123	310
NED (Type III)	160	165	289	356	205	242	278	336	209	190	147	111	267	91	312
VEG (Type III)	160	165	289	356	205	242	278	336	213	188	153	111	267	89	312
TgCatBr5 (Atypical)	160	165	291	356	205	242	278	362	237	174	140	111	265	89	314
VAND (Amazonian)	162	167	291	356	203	242	276	344	217	170	142	113	277	91	308
GUY-CAN-FAM001 (Caribbean 1)	162	165	291	356	205	242	278	342	213	164	142	109	265	87	312

NA: not amplified; Italic genotyping data represent two distinct peaks;

^a^ among the first 8 markers, a minimum of 5 markers allows identifying the strain type.

In İzmir Province, among the 11 wild birds investigated in Çiğli district, nine PCR positive samples were bioassayed and one type III strain was isolated (11.1%; 1/9) (TgBirdTr_Izmir 1). In Konak district of İzmir, among the nine wild bird samples, eight PCR positive samples were bioassayed and three type II strains (TgBirdTr_Izmir 2, 5 and 7) were isolated (37.5%; 3/8). A type III strain (TgBirdTr_Izmir 3) was isolated in Balçova and a type II strain (TgBirdTr_Izmir 4) was isolated in Seferihisar. Among the two samples analyzed in Bergama, one was a mixture of two type II strains (TgBirdTr_Izmir 6) (Fig 1, Table 1).

In Manisa province, among the six wild bird samples analyzed in Salihli District, five PCR positive samples were bioassayed and four strains were isolated (80%; 4/5) in which two were type II (TgBirdTr_Manisa 3 and 6) and one was a mixture of two type II strains (TgBirdTr_Manisa 5), and the remaining was a mixture of type II and III strains (TgBirdTr_Manisa 4). In Manisa central, among the four wild bird samples, three PCR positive samples were bioassayed and a type III strain (TgBirdTr_Manisa 1) was isolated. In Turgutlu, two samples were bioassayed and both were type II strains (TgBirdTr_Manisa 2 and 7).

TgBirdTr_Izmir 6 and TgBirdTr_Manisa 5 were a mix of two strains of genotype II. TgBirdTr_Manisa 4 was a mix of genotypes II and III. These mixture of strains may be due to authentic multiple infection.

**Fig 2:**
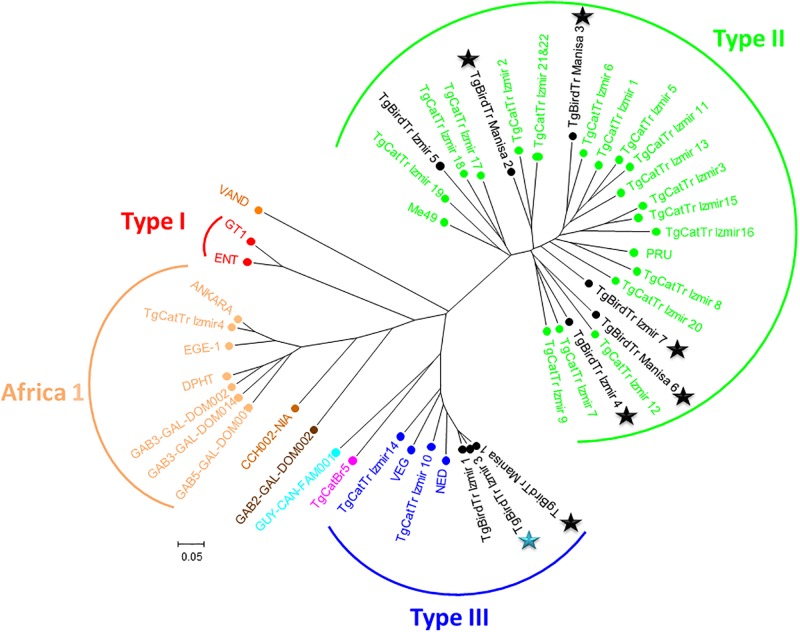
Clustering analysis of 9 *T*. *gondii* strains isolated and genotyped in wild birds of İzmir and Manisa provinces as well as 17 reference *T*. *gondii* strains and 22 *T*. *gondii* strains previously isolated from stray cats of İzmir. Black lettered genotypes represent bird strains isolated in this study. Green lettered genotypes represent type II cat strains previously isolated from İzmir Province and the two type II reference strains (PRU and ME49). Dark blue lettered genotypes represent type III cat strains previously isolated from İzmir Province and the two type III reference strains (VEG and NED). Blue star represents migratory wild bird and black stars represent migratory wild bird with local population.

Cluster analysis showed that among the type II and III *T*. *gondii* strains isolated from wild birds, there is no geographical or species structure when compared with cat strains. Strains isolated from wild birds in in İzmir have close relation with strains isolated from cats in İzmir such as TgBirdTr_İzmir 4 with TgCatTr_İzmir 7&9; TgBirdTr_İzmir 5 with TgCatTr_İzmir 18 (Table 2, Fig 2).

In Manisa province, TgBirdTr_Manisa 2 within type II lineage (have close relation with stray cat strain TgCatTr_İzmir 2, 21 &22. In type II lineage, TgBirdTr_Manisa 6 has close relation with TgCatTr_İzmir 12; TgBirdTr_Manisa 3 has close relation with TgCatTr_İzmir 6 (Table 2, Fig 2).

Wild bird strains within type III lineage which are TgBirdTr_İzmir 1&2 and TgBirdTr_Manisa 1 have close relation with each other and some relation with stray cat strains TgCatTr_İzmir 10 & 14 (Fig 2). Mixed strains and strains with an incomplete genotype (TgBirdTr_Manisa 7 and TgBirdTr_İzmir 2) were not included in the cluster analyses (Table 2, Fig 2).

## Discussion

In this study, the first aim was to show the presence of *T*. *gondii* DNA in wild birds of İzmir and Manisa provinces of Turkey. The prevalence of *Toxoplasma* infection was 89.6% in wild birds based on our molecular screening. A total of 14 strains were genotyped from 43 PCR positive wild birds; Among the 14 strains genotyped, 8 isolates were type II and three isolates were type III. In addition, two samples contained a mix of two type II strains and one was a mixture of type II and type III strain. These mix genotypes which may be due to authentic multiple infections. Nevertheless, these are the first data that show the presence of *T*. *gondii* and genotypes of *T*. *gondii* in wild birds of Turkey.

There are plenty of studies conducted in various wild birds to determine the presence of *T*. *gondii*, isolate *T*. *gondii* strains, genotype them and/or determine seroprevalence using serological and molecular techniques [30–52]. Using serological techniques, *T*. *gondii* antibodies were found in 21–83.3% of various wild birds [31–52]. Among these studies some of them also isolated and genotyped *T*. *gondii* strains.

In Brazil, the seroprevalence in Eared doves (*Zenaida auriculata*) was 22.3% and 12 *T*. *gondii* strains were isolated these doves [36]. In a study conducted in seropositive pigeons of Lisbon Portugal, genotyping of *T*. *gondii* was achieved in 70.7% (29/41) and of the isolates genotyped, 26 samples were type II, two were type III, and one strain was type I [37]. In Slovakia, a total of 10 birds from wild life were examined and the prevalence of *T*. *gondii* DNA was 40% in which type II and III strains were isolated [38]. In New Zeland, *T*. *gondii* was identified in kereru (*Hemiphaga novaeseelandiae*), North Island brown kiwi (*Apteryx mantelli*), and one North Island kaka (*Nestor meridionalis*) and atypical type II genotypes were detected in these birds [39]. In a study conducted in Iran, prevalence of *T*. *gondii* was determined in sparrows (*Passer domesticus*), pigeons (*Columba livia*) and starlings (*Sturnus vulgaris*) and *T*. *gondii* DNA was detected in 26.5% (n: 64), 6.9% (n:43), and 12.8% (n:39), respectively [41]. In China, *T*. *gondii* was investigated in 178 wild birds including Common pheasants (*Phasianus colchicus*), Tree sparrows (*Passer montanus*), House sparrows (*Passer domesticus*), Saxaul sparrows (*Passer ammodendri*), Cinnamon sparrow (*Passer rutilans*), and four of them were *T*. *gondii* DNA positive in which they were type I and II strains [42]. In the USA, the seropositivity rate in 632 Mute swans (*Cygnus olor*) was 8.5% and three strains were genotyped from seropositive 14 swans which were Type III and a new genotype [44]. In Colorado State of USA, 38 of 382 wild birds were seropositive (9.9%) and viable *T*. *gondii* was isolated from barn owl (*Tyto alba*), American kestrels (*Falco sparverius*), Ferruginous hawks (*Buteo regalis*), Roughlegged buzzard (*Buteo lagopus*), Swainson’s hawks (*Buteo swainsoni*), and Red-tailed hawks (*Buteo jamaicensis*) [45]. In France, *T*. *gondii* type II strain was isolated from common mallards (*Anas platyrhynchos*) [46]. *T*. *gondii* was also identified in various wild birds such as a Bald Eagle (*Haliaeetus leucocephalus*), bar-shouldered dove (*Geopelia humeralis*), Great Spotted Woodpecker (*Dendrocopos major*), Amazon Parrot (*Amazona vinacea*), red-shouldered hawk (*Buteo lineatus*), bald eagle (*Haliaeetus leucocephalus*) [48–52].

*T*. *gondii* studies in birds carried out in Turkey are limited. The seroprevalence of *T*. *gondii* in pigeons (*Columba livia livia*) was 0.95% using Sabin Feldman dye test in Niğde province located in central Anatolia [53]. In Kayseri province located in central Anatolia, a total of 44 birds from wild life were examined with Sabin Feldman dye test and the seroprevalence of *T*. *gondii* was 40% [54]. In a study conducted in Hatay province located in southern Anatolia and Van province located in eastern Anatolia, *T*. *gondii* DNA was detected in seven wild avian species among 103 wild birds (6.79%), comprising 20 species [55].

Globally, the presence of *T*. *gondii* range between 21–83.3% in wild birds using serological analyses or PCR, and 89.6% in wild birds of prey of İzmir and Manisa provinces using PCR in this study. This high prevalence is possibly due to eating habits of wild birds which prey for intermediate hosts of *T*. *gondii*. Wild birds can also be infected by water sources contaminated with *T*. *gondii* oocysts or drinking seawater since *T*. *gondii* oocyst can sporulate and survive in seawater [56].

There were several causes of death in the group of wild birds analyzed in this study which were mostly trauma. In 16.7% of PCR positive wild birds, symptoms such as paralyses (n: 6) and incoordination of muscles (n: 1) were observed possibly suggestive of toxoplasmosis.

Analyses the presence of *Toxoplasma* infection in predator wild birds (n: 39) except the ones eating fish, insects, reptiles, and small invertebrates showed that 92.3% of them contained *T*. *gondii* DNA in their tissues. In the remaining 9 birds that are not predator or eat fish, insects, reptiles, and small invertebrates, the prevalence of *T*. *gondii* DNA was 88.9%. The *Toxoplasma* infection rate in both groups is pretty high. This can be anticipated for predator birds since they commonly feed with the rodent intermediate which can host of *T*. *gondii*. The high prevalence in other birds shows that oocysts may represent a significant source of contamination for them, via contaminated water or soil [56].

In this study, a total of 14 strains were genotyped from 43 PCR positive wild birds. Among these 14 strains genotyped, 8 isolates were type II, three isolates were type III, and in addition, two samples contained a mix of two type II strains and one was a mixture of type II and type III strain. Any African genotype was not detected. These findings can be analyzed in terms of migration specifications (local residents, partially migratory, and migratory). The prevalence of *T*. *gondii* DNA in the migratory birds (n: 43) is 88.4% and 80% in the local resident wild bird group (n: 5). The *Toxoplasma* infection rate in both groups is again pretty high. Genotypes II and III infection were detected in both populations as could be expected from previous results obtained in cat population from Turkey [17, 22]. Genotype II and type III are highly prevalent in Europe, with more type III in southern Europe. They are also prevalent in Mediterranean countries [57].

Distance genetic tree that was built only with strains identified with at least 14 MS markers included nearly only strains isolated from migratory birds (except TgBirdTr_Izmir 1 & 5) and those were close to strains isolated from strain cats in Izmir. It may be hard to draw a conclusion that migratory birds have a role to disseminate *T*. *gondii* genotypes to stray cats based on these results. On the contrary, oocysts excreted in the environment by stray cats could be the direct or indirect source of contamination of wild birds.

Cluster analysis showed that *T*. *gondii* strains isolated from birds in İzmir and Manisa within type II and III lineage have close relation with strains isolated from cats in İzmir (Table 2, Fig 2). This could be due to the small distance between these areas. In addition, when we analyze the location of the PCR positive wild birds, a small motion area appears in Gediz lowland between İzmir and Manisa provinces (represented by dotted lane in Fig 1) possibly due to feeding habits of wild birds. This motion area is connected to İzmir Bird Sanctuary which is a popular stop point for resident and migratory birds of prey. Both type II and III strains are isolated in Çiğli district where the İzmir Bird Sanctuary is located and type II and III strains are also isolated on this route.

As Africa 1 was detected in a cat and two human cases previously, we expected to find it in migratory wild birds as an explanation of its presence in Turkey. This was not the case. This may be due to the inexistence of Africa 1 in the wild birds species included to this study or Africa 1 may have been transferred to Turkey via other routes such as rodents prevalent in trade ships. This could be an explanation of the absence of detection of African genotypes in our sampling. More sampling from different host species and areas would be needed to answer this question.

## Conclusion

Overall, the two major clonal lineages (type II and III) have been isolated for the first time from wild birds in İzmir and Manisa provinces of Turkey. There can be a probable route of transmission between stray cats and wild birds of İzmir and Manisa based on cluster analyses. Further studies are required to isolate more strains from human cases, other intermediate hosts, and water sources to reveal this relation.

## References

[1] Weiss LM, Kim K. *Toxoplasma gondii*, The Model Apicomplexan: Perspectives and Methods. Great Britain: Elsevier Ltd; 2007.

[2] Dardé ML. *Toxoplasma gondii*, "new" genotypes and virulence. Parasite. 2008; 15(3):366–71. doi: 10.1051/parasite/2008153366 18814708 10.1051/parasite/2008153366

[3] Ajzenberg D, Yera H, Marty P, Paris L, Dalle F, Menotti J, et al Genotype of 88 *Toxoplasma gondii* isolates associated with toxoplasmosis in immunocompromised patients and correlation with clinical findings. J Infect Dis. 2009; 199(8):1155–1167. doi: 10.1086/597477 19265484 10.1086/597477

[4] Delhaes L, Ajzenberg D, Sicot B, Bourgeot P, Dardé ML, Dei-Cas E, et al Severe congenital toxoplasmosis due to a *Toxoplasma gondii* strain with an atypical genotype: case report and review. Prenat Diagn. 2010; 30(9):902–905. doi: 10.1002/pd.2563 20582922 10.1002/pd.2563

[5] Ajzenberg D, Cogné N, Paris L, Bessières MH, Thulliez P, Filisetti D, et al Genotype of 86 *Toxoplasma gondii* isolates associated with human congenital toxoplasmosis, and correlation with clinical findings. J Infect Dis. 2002; 186(5):684–689. doi: 10.1086/342663 12195356 10.1086/342663

[6] Lorenzi H, Khan A, Behnke MS, Namasivayam S, Swapna LS, Hadjithomas M, et al Local admixture of amplified and diversified secreted pathogenesis determinants shapes mosaic *Toxoplasma gondii* genomes. Nat Commun. 2016; 7:10147 doi: 10.1038/ncomms10147 26738725 10.1038/ncomms10147 PMC4729833

[7] Shwab EK, Zhu XQ, Majumdar D, Pena HF, Gennari SM, Dubey JP, et al Geographical patterns of *Toxoplasma gondii* genetic diversity revealed by multilocus PCR-RFLP genotyping. Parasitology. 2014; 141(4):453–461. doi: 10.1017/S0031182013001844 24477076 10.1017/S0031182013001844

[8] Bontell IL, Hall N, Ashelford KE, Dubey JP, Boyle JP, Lindh J, et al Whole genome sequencing of a natural recombinant *Toxoplasma gondii* strain reveals chromosome sorting and local allelic variants. Genome Biol. 2009; 10(5):R53 doi: 10.1186/gb-2009-10-5-r53 19457243 10.1186/gb-2009-10-5-r53 PMC2718519

[9] Mercier A, Devillard S, Ngoubangoye B, Bonnabau H, Bañuls AL, Durand P, et al Additional haplogroups of *Toxoplasma gondii* out of Africa: population structure and mouse-virulence of strains from Gabon. PLoS Negl Trop Dis. 2010; 4(11):e876 doi: 10.1371/journal.pntd.0000876 21072237 10.1371/journal.pntd.0000876 PMC2970538

[10] Al-Kappany YM, Rajendran C, Abu-Elwafa SA, Hilali M, Su C, Dubey JP. Genetic diversity of *Toxoplasma gondii* isolates in Egyptian feral cats reveals new genotypes. J Parasitol. 2010; 96(6):1112–1114. doi: 10.1645/GE-2608.1 21158618 10.1645/GE-2608.1

[11] Dubey JP, Pas A, Rajendran C, Kwok OC, Ferreira LR, Martins J, et al Toxoplasmosis in Sand cats (*Felis margarita*) and other animals in the Breeding Centre for Endangered Arabian Wildlife in the United Arab Emirates and Al Wabra Wildlife Preservation, the State of Qatar. Vet Parasitol. 2010; 172(3–4):195–203. doi: 10.1016/j.vetpar.2010.05.013 20570441 10.1016/j.vetpar.2010.05.013 PMC7116901

[12] Salant H, Weingram T, Spira DT, Eizenberg T. An outbreak of Toxoplasmosis amongst squirrel monkeys in an Israeli monkey colony. Vet Parasitol. 2009; 159(1):24–29. doi: 10.1016/j.vetpar.2008.10.011 19019554 10.1016/j.vetpar.2008.10.011

[13] Wang L, Chen H, Liu D, Huo X, Gao J, Song X, et al Genotypes and Mouse Virulence of *Toxoplasma gondii* Isolates from Animals and Humans in China. PLoS One. 2013; 8(1):e53483 doi: 10.1371/journal.pone.0053483 23308233 10.1371/journal.pone.0053483 PMC3538538

[14] Zhou Y, Zhang H, Cao J, Gong H, Zhou J. Isolation and genotyping of *Toxoplasma gondii* from domestic rabbits in China to reveal the prevalence of type III strains. Vet Parasitol. 2013; 193(1–3):270–276. 23261088 10.1016/j.vetpar.2012.11.031

[15] Dubey JP, Huong LT, Lawson BW, Subekti DT, Tassi P, Cabaj W, et al Seroprevalence and isolation of *Toxoplasma gondii* from free-range chickens in Ghana, Indonesia, Italy, Poland, and Vietnam. J Parasitol. 2008; 94(1):68–71. doi: 10.1645/GE-1362.1 18372623 10.1645/GE-1362.1

[16] Zia-Ali N, Fazaeli A, Khoramizadeh M, Ajzenberg D, Dardé M, Keshavarz-Valian H. Isolation and molecular characterization of *Toxoplasma gondii* strains from different hosts in Iran. Parasitol Res. 2007; 101(1):111–115. doi: 10.1007/s00436-007-0461-7 17333278 10.1007/s00436-007-0461-7

[17] Chaichan P, Mercier A, Galal L, Mahittikorn A, Ariey F, Morand S, et al Geographical distribution of *Toxoplasma gondii* genotypes in Asia: A link with neighboring continents. Infect Genet Evol. 2017; 53:227–238. doi: 10.1016/j.meegid.2017.06.002 28583867 10.1016/j.meegid.2017.06.002

[18] Khan A, Dubey JP, Su C, SibleyL D, Ajioka JW, Rosenthal BM. Genetic analyses of atypical *Toxoplasma gondii* strains reveal a fourth clonal lineage in North America. Int J Parasitol. 2011; 41(6):645–655. doi: 10.1016/j.ijpara.2011.01.005 21320505 10.1016/j.ijpara.2011.01.005 PMC3081397

[19] Rajendran C, Su C, Dubey JP. Molecular genotyping of *Toxoplasma gondii* from Central and South America revealed high diversity within and between populations. Infect Genet Evol. 2012; 12(2):359–368. doi: 10.1016/j.meegid.2011.12.010 22226702 10.1016/j.meegid.2011.12.010

[20] Pena HF, Gennari SM, Dubey JP, Su C. Population structure and mouse-virulence of *Toxoplasma gondii* in Brazil. Int J Parasitol. 2008; 38(5):561–569. 17963770 10.1016/j.ijpara.2007.09.004

[21] Döşkaya M, Caner A, Ajzenberg D, Değirmenci A, Dardé ML, Can H, et al Isolation of *Toxoplasma gondii* strains similar to Africa 1 genotype in Turkey. Parasitol Int. 2013; 62(5): 471–474. doi: 10.1016/j.parint.2013.06.008 23811201 10.1016/j.parint.2013.06.008

[22] Can H, Döşkaya M, Ajzenberg D, Özdemir HG, Caner A, İz SG, et al Genetic characterization of *Toxoplasma gondii* isolates and toxoplasmosis seroprevalence in stray cats of İzmir, Turkey. PLoS One. 2014; 9(8):e104930 doi: 10.1371/journal.pone.0104930 eCollection 2014. 25127360 10.1371/journal.pone.0104930 PMC4134241

[23] The International Union for Conservation of Nature and Natural Resources (IUCN) Red List of Threatened Species. Accession date: 22.03.2018. http://www.iucnredlist.org/

[24] Döşkaya M, Caner A, Can H, İz SG, Değirmenci A, Gürüz AY. Cryopreservation of *Toxoplasma gondii* tachyzoites and tissue cysts. Turkiye Parazitol Derg. 2013; 37(1):44–46. 23619046 10.5152/tpd.2013.11

[25] Cassaing S, Bessières MH, Berry A, Berrebi A, Fabre R, Magnaval JF. Comparison between two amplification sets for molecular diagnosis of toxoplasmosis by real-time PCR. J Clin Microbiol. 2006; 44(3):720–4. doi: 10.1128/JCM.44.3.720-724.2006 16517845 10.1128/JCM.44.3.720-724.2006 PMC1393120

[26] Ajzenberg D, Collinet F, Mercier A, Vignoles P, Dardé ML. Genotyping of *Toxoplasma gondii* isolates with 15 microsatellite markers in a single multiplex PCR assay. J Clin Microbiol. 2010; 48(12): 4641–4645. doi: 10.1128/JCM.01152-10 20881166 10.1128/JCM.01152-10 PMC3008440

[27] Mercier A, Ajzenberg D, Devillard S, Demar MP, de Thoisy B, Bonnabau H, et al Human impact on genetic diversity of *Toxoplasma gondii*: example of the anthropized environment from French Guiana. Infect Genet Evol. 2011; 11(6): 1378–1387. doi: 10.1016/j.meegid.2011.05.003 21600306 10.1016/j.meegid.2011.05.003

[28] Su C, Khan A, Zhou P, Majumdar D, Ajzenberg D, Dardé ML, et al Globally diverse *Toxoplasma gondii* isolates comprise six major clades originating from a small number of distinct ancestral lineages. Proc Natl Acad Sci U S A. 2012; 109(15):5844–5849. doi: 10.1073/pnas.1203190109 22431627 10.1073/pnas.1203190109 PMC3326454

[29] Cavalli-Sforza LL, Edwards AW. Phylogenetic analysis. Models and estimation procedures. Am J Hum Genet. 1967; 19(3 Pt 1):233–257. 6026583 PMC1706274

[30] Dubey JP. A review of toxoplasmosis in wild birds. Vet Parasitol. 2002; 106(2):121–153. 12031816 10.1016/s0304-4017(02)00034-1

[31] Tidy A, Fangueiro S, Dubey JP, Cardoso L, Lopes AP. Seroepidemiology and risk assessment of *Toxoplasma gondii* infection in captive wild birds and mammals in two zoos in the North of Portugal. Vet Parasitol. 2017; 235:47–52. 28215867 10.1016/j.vetpar.2017.01.004

[32] Gennari SM, Niemeyer C, Soares HS, Musso CM, Siqueira GC, Catão-Dias JL, et al Seroprevalence of *Toxoplasma gondii* in seabirds from *Abrolhos Archipelago*, Brazil. Vet Parasitol. 2016a; 226:50–2. 27514883 10.1016/j.vetpar.2016.06.016

[33] Gennari SM, Niemeyer C, Catão-Dias JL, Soares HS, Acosta IC, Dias RA, et al Survey of *Toxoplasma gondii* antibodies in Magellanic Penguins (*Spheniscus Magellanicus Forster*, 1781). J Zoo Wildl Med. 2016b; 47(1):364–6. doi: 10.1638/2015-0103.1 27010304 10.1638/2015-0103.1

[34] Work TM, Verma SK, Su C, Medeiros J, Kaiakapu T, Kwok OC, et al *Toxoplasma gondii* antibody prevalence and two new genotypes of the parasite in endangered Hawaiian geese (nene: *Branta sandvicensis*). J Wildl Dis. 2016; 52(2):253–257. doi: 10.7589/2015-09-235 26967138 10.7589/2015-09-235

[35] Straub MH, Kelly TR, Rideout BA, Eng C, Wynne J, Braun J, et al Seroepidemiologic Survey of Potential Pathogens in Obligate and Facultative Scavenging Avian Species in California. PLoS One. 2015; 10(11):e0143018 doi: 10.1371/journal.pone.0143018 eCollection 2015. 26606755 10.1371/journal.pone.0143018 PMC4659623

[36] Barros LD, Taroda A, Zulpo DL, Cunha IA, Sammi AS, Cardim ST, et al Genetic characterization of *Toxoplasma gondii* isolates from eared doves (*Zenaida auriculata*) in Brazil. Rev Bras Parasitol Vet. 2014; 23(4):443–448. doi: 10.1590/S1984-29612014073 25517521 10.1590/S1984-29612014073

[37] Vilares A, Gargaté MJ, Ferreira I, Martins S, Júlio C, Waap H, et al Isolation and molecular characterization of *Toxoplasma gondii* isolated from pigeons and stray cats in Lisbon, Portugal. Vet Parasitol. 2014; 205(3–4):506–511. doi: 10.1016/j.vetpar.2014.08.006 25195193 10.1016/j.vetpar.2014.08.006

[38] Turčeková Ľ, Hurníková Z, Spišák F, Miterpáková M, Chovancová B. *Toxoplasma gondii* in protected wildlife in the Tatra National Park (TANAP), Slovakia. Ann Agric Environ Med. 2014;21(2):235–238. doi: 10.5604/1232-1966.1108582 24959767 10.5604/1232-1966.1108582

[39] Howe L, Hunter S, Burrows E, Roe W. Four cases of fatal toxoplasmosis in three species of endemic New Zealand birds. Avian Dis. 2014; 58(1):171–175. doi: 10.1637/10625-080413-Case.1 24758132 10.1637/10625-080413-Case.1

[40] Gennari SM, Ogrzewalska M, Soares HS, Saraiva DG, Pinter A, Labruna MB, et al Occurrence of *Toxoplasma gondii* antibodies in birds from the Atlantic Forest, state of São Paulo, Brazil. Vet Parasitol. 2014; 200(1–2):193–197. doi: 10.1016/j.vetpar.2013.10.003 24332961 10.1016/j.vetpar.2013.10.003

[41] Khademvatan S, Saki J, Yousefi E, Abdizadeh R. Detection and genotyping of *Toxoplasma gondii* strains isolated from birds in the southwest of Iran. Br Poult Sci. 2013;54(1):76–80. doi: 10.1080/00071668.2013.763899 23444856 10.1080/00071668.2013.763899

[42] Huang SY, Cong W, Zhou P, Zhou DH, Wu SM, Xu MJ, et al First report of genotyping of *Toxoplasma gondii* isolates from wild birds in China. J Parasitol. 2012; 98(3):681–682. doi: 10.1645/GE-3038.1 22263675 10.1645/GE-3038.1

[43] Cabezón O, García-Bocanegra I, Molina-López R, Marco I, Blanco JM, Höfle U, et al Seropositivity and risk factors associated with *Toxoplasma gondii* infection in wild birds from Spain. PLoS One. 2011; 6(12):e29549 doi: 10.1371/journal.pone.0029549 22216311 10.1371/journal.pone.0029549 PMC3245288

[44] Dubey JP, Choudhary S, Kwok OC, Ferreira LR, Oliveira S, Verma SK, et al Isolation and genetic characterization of *Toxoplasma gondii* from mute swan (*Cygnus olor*) from the USA. Vet Parasitol. 2013; 195(1–2):42–46. 23394800 10.1016/j.vetpar.2012.12.051

[45] Dubey JP, Felix TA, Kwok OC. Serological and parasitological prevalence of *Toxoplasma gondii* in wild birds from Colorado. J Parasitol. 2010; 96(5):937–939. doi: 10.1645/GE-2501.1 20950101 10.1645/GE-2501.1

[46] Aubert D, Ajzenberg D, Richomme C, Gilot-Fromont E, Terrier ME, de Gevigney C, et al Molecular and biological characteristics of *Toxoplasma gondii* isolates from wildlife in France. Vet Parasitol. 2010; 171(3–4):346–349. doi: 10.1016/j.vetpar.2010.03.033 20417034 10.1016/j.vetpar.2010.03.033

[47] Uterak I, Hejlicek K, Nezval J, Folk C. Incidence of *Toxoplasma gondii* in populations of wild birds in the Czech Republic. Avian Pathol. 1992; 21(4):659–665. doi: 10.1080/03079459208418887 18670984 10.1080/03079459208418887

[48] Szabo KA, Mense MG, Lipscomb TP, Felix KJ, Dubey JP. Fatal toxoplasmosis in a bald eagle (*Haliaeetus leucocephalus*). J Parasitol. 2004; 90(4):907–908. doi: 10.1645/GE-270R 15357102 10.1645/GE-270R

[49] Rigoulet J, Hennache A, Lagourette P, George C, Longeart L, Le Net JL, et al Toxoplasmosis in a bar-shouldered dove (*Geopelia humeralis*) from the Zoo of Clères, France. Parasite. 2014;21:62 doi: 10.1051/parasite/2014062 25407506 10.1051/parasite/2014062 PMC4236686

[50] Jokelainen P, Vikøren T. Acute fatal toxoplasmosis in a Great Spotted Woodpecker (*Dendrocopos major*). J Wildl Dis. 2014; 50(1):117–120. doi: 10.7589/2013-03-057 24171576 10.7589/2013-03-057

[51] Ferreira FC Jr, Donatti RV, Marques MV, Ecco R, Preis IS, Shivaprasad HL, et al Fatal toxoplasmosis in a vinaceous Amazon parrot (*Amazona vinacea*). Avian Dis. 2012; 56(4):774–777. doi: 10.1637/10063-011912-Case.1 23397856 10.1637/10063-011912-Case.1

[52] Yu L, Shen J, Su C, Sundermann CA. Genetic characterization of *Toxoplasma gondii* in wildlife from Alabama, USA. Parasitol Res. 2013; 112(3):1333–1336. doi: 10.1007/s00436-012-3187-0 23160892 10.1007/s00436-012-3187-0

[53] Karatepe M, Kılıç S, Karatepe B, Babür C. Prevalence of *Toxoplasma gondii* antibodies in domestic (*Columba livia domestica*) and wild (*Columba livia livia*) pigeons in Niğde region, Turkey. Turkiye Parazitol Derg. 2011;35(1):23–26. doi: 10.5152/tpd.2011.06 21618187 10.5152/tpd.2011.06

[54] İnci A, Babür C, Çam Y, İça A. Investigation of Seropositivity of *Toxoplasma gondii* (Nicolle and Manceaux, 1908) in some Prey Birds. F Ü Sağlık Bil Derg. 2002; 16(2):177–179.

[55] Muz MN, Kılınç ÖO, İșler CT, Altuğ E, Karakavuk M. Molecular diagnosis of *Toxoplasma gondii* and *Neospora caninum* in brain tissues of some wild birds. Kafkas Üniversitesi Veteriner Fakültesi Dergisi. 2014; 21(2), 173–178.

[56] Lindsay DS, Collins MV, Mitchell SM, Cole RA, Flick GJ, Wetch CN, et al Sporulation and survival of *Toxoplasma gondii* oocysts in seawater. J Eukaryot Microbiol. 2003;50 Suppl:687–8. 14736220 10.1111/j.1550-7408.2003.tb00688.x

[57] Galal L, Ajzenberg D, Hamidović A, Durieux MF, Dardé ML, Mercier A. Toxoplasma and Africa: One Parasite, Two Opposite Population Structures. Trends Parasitol. 2018; 34(2):140–154. doi: 10.1016/j.pt.2017.10.010 29174610 10.1016/j.pt.2017.10.010

